# Navigating the treatment landscape of Alzheimer's disease: Current strategies and future directions

**DOI:** 10.1002/ibra.12197

**Published:** 2025-05-10

**Authors:** Tapas Kumar Mohapatra, Reena Rani Nayak, Ankit Ganeshpurkar, Prashant Tiwari, Dileep Kumar

**Affiliations:** ^1^ Department of Pharmacology Nityananda College of Pharmacy Seragarh Balasore Odisha India; ^2^ Department of Pharmaceutical Chemistry Nityananda College of Pharmacy Balasore Odisha India; ^3^ Department of Pharmaceutical Sciences Dr. Harisingh Gour Vishwavidyalaya (A Central University) Sagar Madhya Pradesh India; ^4^ Department of Pharmacology Dayanandasagar University Bangalore Karnataka India; ^5^ Department of Pharmaceutical Chemistry, Manipal College of Pharmaceutical Sciences Manipal Academy of Higher Educatio (MAHE) Manipal Karnataka India

**Keywords:** Alzheimer's disease, neurofibrillary tangles, N‐methyl‐D aspartate receptors, presenilin, β‐amyloid

## Abstract

Alzheimer's disease (AD), a neurodegenerative disease leading to dementia, lacks a single definitive diagnosis. While current medications only manage symptoms, the ideal treatment would restore cognition. Traditional therapies targeting beta‐amyloid haven't yielded significant results, while new approaches target tau protein tangles, protein degradation pathways, inflammation, and neurotrophic factor depletion. Autophagy, a cellular degradation and recycling process, has emerged as a crucial hallmark and contributor to the pathogenesis of AD. Notably, autophagy induction has emerged as a promising therapeutic approach, with inducers like celastrol and caudatin promoting the degradation of toxic protein aggregates. Additionally, innovative drug formulations, such as nanoparticles, are being explored for targeted drug delivery. Research is increasingly focusing on neuroinflammation and developing multi‐targeted drugs to address various aspects of AD, potentially leading to preventive strategies in the early stages. This review summarizes the current state and emerging trends in AD drug development.

## Graphical abstract

Alzheimer's disease treatment is evolving rapidly, with emerging strategies targeting multiple disease pathways. This graphical abstract highlights the shift from traditional therapies to innovative approaches that hold promise for improving outcomes.

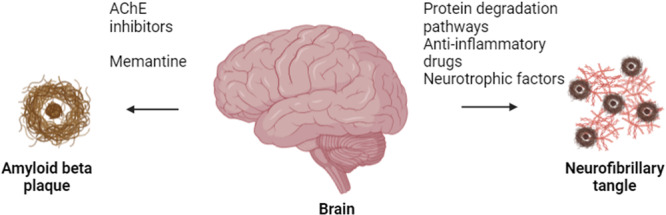

## 1 INTRODUCTION

Alzheimer's disease (AD) is dementia caused by degeneration of the brain. It is the most common cause of dementia in the elderly population accounting for 60% to 80% and leads to intellectual disabilities. It is a slow progressive disease leading to neuronal loss characterized by memory problems, impaired judgment, speech impairment, and behavioral changes.^1^ There are two primary classifications of AD, in which the initial classification pertains to hereditary or autosomal dominant AD, which manifests before the age of 65, affecting approximately 1% of patients, while the secondary classification encompasses sporadic AD, which affects individuals aged over 65 and is contingent upon age.^2^ In 2015, it was estimated that 46.8 million individuals globally suffered from a familial history of AD. This figure is projected to be raised to 131.5 million by 2050, thereby exacerbating associated health and financial implications.^3^ At the early stages of AD, individuals frequently experiencing a decline in their memory function. This decline can be attributed to the malfunctioning of the hippocampus, a critical brain structure that plays a pivotal role in memory formation. As the hippocampus deteriorates it becomes difficult to retrieve old memories along with laying down new memories.^4^ In the mid‐stage of AD, the frontal lobe gets progressively compromised leading to difficulty in managing daily routines along with increased confusion and disorientation.^5^ Additionally, the damage to the limbic system, including the amygdala, translates to personality and behavioral changes leading to the development of apathy, depression, or social withdrawal.^6^ The deterioration also extends to the parietal lobe, responsible for spatial processing and motor coordination. Consequently, mid‐stage AD patients may struggle with spatial tasks and navigation.^7^ The unbridled progression of AD in its late stages causes widespread brain atrophy, and levy havoc on cognitive and physical functionalities. This extensive neurodegeneration manifests as a severe global decline, encompassing memory, language, and reasoning abilities. The pathological onslaught progresses towards the brainstem and other critical regions, leading to physical impairments that include compromised balance, coordination difficulties, swallowing problems, and loss of bladder control.^8^ The urgency of developing a meaningful therapy for AD is undeniable, considering the devastating impact it has on the cognition and physical abilities of patients.

There are two pathophysiological changes associated with AD, that is, extracellularly located abnormal amyloid β (Aβ) plaques and intracellular hyperphosphorylated tau protein‐producing neurofibrillary tangles (NFTs) within neurons.^9^, ^10^ Local dysfunction of dendrites, axons, and synapses, characterized by impaired signal transmission, results from the initial oligomerization of soluble β‐amyloid into Aβ plaques in the brain. Previous studies have focused on pathological components of Aβ, particularly soluble Aβ oligomers (AβO), which are implicated in the neuropathology of AD and can be detected even before the first symptoms appear.^11^, ^12^, ^13^, ^14^ In the absence of effective treatment, AβO gradually accumulates, forming lesions such as plaques or tangles that correspond to areas of neuron loss in the brain.^15^

**FIGURE 1:**
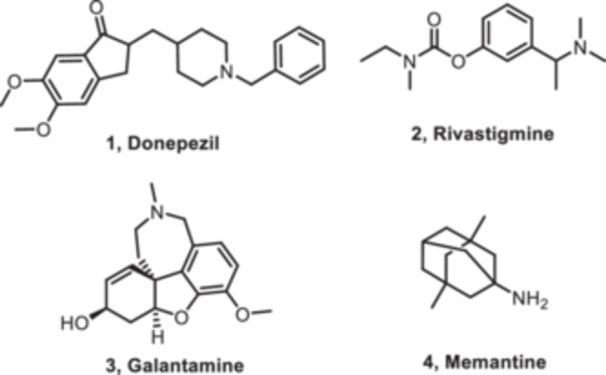
FDA‐approved drugs for the treatment of AD. These drugs focus on alleviating symptoms and slowing down cognitive decline. AD, Alzheimer's disease.

The therapeutic regime for the treatment of AD currently comprises four approved drugs that are effective against the symptoms. These medications belong to two distinct pharmacological classes: acetylcholinesterase (AChE) inhibitors and N‐methyl‐d‐aspartate (NMDA) receptor antagonists. AChE inhibitors, including donepezil (**1**), rivastigmine (**2**), and galantamine (**3**), reduce the enzymatic turnover of acetylcholine (ACh) degradation within the synaptic cleft, thereby boosting cholinergic neurotransmission.^16^ This increase in ACh is believed to be responsible for some improvement in thinking and memory in AD patients. NMDA receptor antagonists, exemplified by memantine (**4**), function by modulating glutamatergic excitotoxicity^17^ (Figure 1). Glutamate, an excitatory neurotransmitter, is believed to play a role in the neurodegenerative processes associated with AD. Hence, inhibition of NMDA receptors by memantine may mitigate the deleterious effects of excessive glutamate signaling.

Despite the established benefits of these medications in enhancing cognitive function and patient quality of life, their therapeutic impact remains moderate. The precise etiology of AD remains elusive, posing a significant challenge to the development of truly preventative or curative interventions. This underscores the continuous need for persistent research aimed at elucidating the underlying pathophysiology of AD and identifying novel therapeutic targets. This review explores the limitations of these approaches and emphasizes the need for new therapies that target the root causes. The potential of repurposing existing interventions and exploring novel avenues for treating AD will also be discussed. The novelty of this review lies in shifting focus from symptomatic treatments to disease‐modifying approaches, offering new insights into more effective and faster therapeutic solutions.

## 2 RISK FACTORS FOR AD

Understanding the factors that increase a person's risk of developing AD is crucial for early detection, prevention strategies, and the development of effective treatments.

### 2.1 Health and lifestyle factors

Cardiovascular health plays a significant role in AD risk. Conditions like obesity, hypertension, high cholesterol, and diabetes are all associated with an increased risk of developing AD. The exact connection between these conditions and AD remains under investigation. However, the existing evidence suggests that cardiovascular disease, a common thread in these conditions, might contribute to dementia onset due to shared genetic and risk factors. People are advised to maintain a healthy lifestyle such as quitting smoking, restricting alcohol consumption and managing stress.^18^ Healthy eating habits, a balanced diet, exercising physically and mentally, and constantly monitoring their health are some important practices that are needed to prevent AD.

### 2.2 Environmental factors

Exposure to heavy metals and pesticides is reported to cause mental decline and neurodegenerative diseases. Evidence suggests that exposure to air pollution and fungal infections are associated with the development of AD.^19^, ^20^ Metal rich magnetic derived nanoparticles, combustion products present in the air are found oxidative and associated with mitochondrial dysfunction. Unfolded protein, calcium homeostasis, and apoptotic signaling are all known features of AD caused due to these factors^21^.

### 2.3 Genetic factors

Studies have shown that patients with certain medical conditions have a higher risk of developing AD. People with Down syndrome, a genetic disorder, cause amyloid plaque formation in the brain over time, leading to AD.^22^ Familial AD (FAD) is associated with earlier symptoms in patients at the age of 30 or 40 s and accounts for approximately 23% of AD patients.^23^ Frequent mutation in the presenilin1 (PSEN1) and presenilin 2 (PSEN2) genes, which encode the two subunits of γ secretase is also a cause of AD.^24^ Homozygous apolipoprotein E4 (ApoE4) has been identified as the biggest risk factor, increasing the AD risk up to 8 times.^25^

### 2.4 Other crucial factors

**FIGURE 2:**
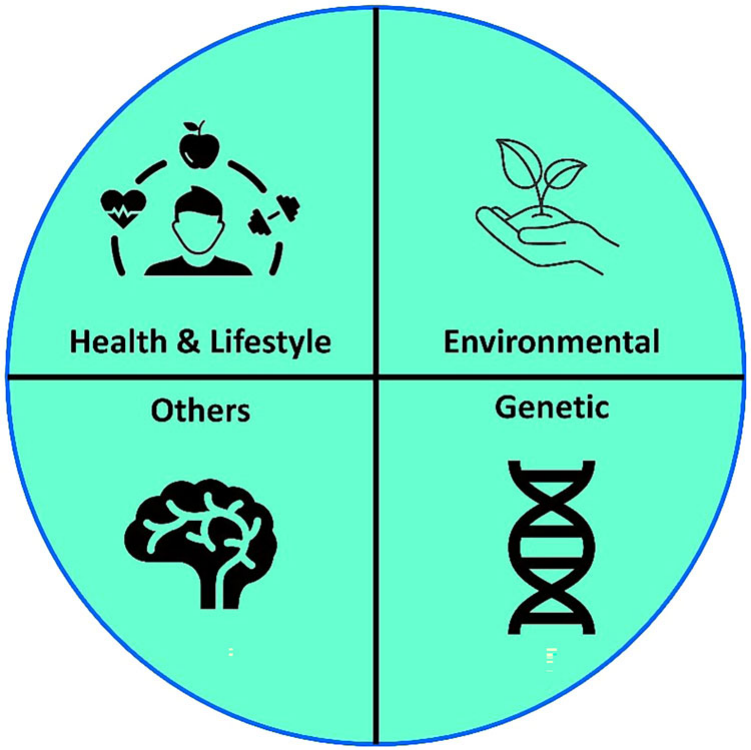
Risk factors for Alzheimer's disease (AD). This figure highlights the key factors that increase the risk of developing AD. These include environmental factors, genetic predisposition (e.g., the presence of the APOE4 gene), lifestyle habits (such as poor diet and lack of physical activity), and medical conditions like high blood pressure, diabetes, and heart disease. [Color figure can be viewed at wileyonlinelibrary.com]

Abnormal metabolism of glucose and lipids, neuronal inflammation, cerebrovascular abnormalities and blockade of endosomal pathways can also contribute to the pathology of AD in the brain.^26^, ^27^, ^28^ The vascular system becomes impaired and fails to deliver sufficient blood and nutrients to the brain and clear away the debris of metabolic products, leading to chronic inflammation by the activation of astrocytes and microglia ^29^ (Figure 2).

## 3 PATHOPHYSIOLOGY AND NEUROLOGICAL FEATURES OF AD

The classical hallmarks of the pathophysiology of AD are Aβ plaques and NFTs, which are responsible for the notable structural and functional deterioration of the central nervous system (CNS). Abnormal aggregation and accumulation of the Aβ peptide in the extracellular space outside the neurons results in the formation of Aβ plaques. Amyloid precursor protein (APP) is a protein implicated in neurite repair, cell communication, and maintaining brain structure.^30^ Enzymatic processing of APP at specific sites results in fragments with multiple functions. The cleavage of APP by α‐secretase within the Aβ domain is the preferred “non‐amyloidogenic pathway,” which prevents the formation of the neurotoxic Aβ peptide.^31^ The generated fragments are beneficial in promoting neurite repair and cellular signaling. In contrast, the other “amyloidogenic pathway” is implicated in AD. It involves initial cleavage through β‐secretase and subsequent processing by γ‐secretase, leading to Aβ formation. Aβ aggregation into plaques disrupts brain function and is a hallmark of AD pathology.^32^ Thus, the specific enzymatic pathway governing APP processing determines its influence on neuronal health and disease development. In healthy individuals, these initial monomers are typically cleared from the brain. If not cleared, multiple Aβ monomers can aggregate together, forming small, soluble aggregates called oligomers with the potential to disrupt neuronal communication and impair their function. Oligomers can further aggregate into less soluble elongated, thread‐like structures called protofibrils in brain tissue. Protofibrils can mature into fibrils that produce Aβ plaques.^33^ It blocks synaptic function, triggering chronic inflammation and oxidative stress, resulting in neurodegeneration.^34^ The initial deposition of Aβ plaques occurs in a diffuse pattern within the neocortex. This is followed by sequential involvement of the hippocampus, subcortical nuclei, and ultimately, the cerebellum.^35^ Fe65 protein is an adaptor protein involved in various cellular processes, including gene regulation and neuronal signaling. In AD brains, APP causes its intracellular part (AICD) to bind with Fe65 protein. This interaction promotes Aβ release, driving AD progression.^36^

Apolipoprotein E (ApoE) is a crucial lipid‐binding protein synthesized in the liver and the brain. It facilitates lipid metabolism and transport in the CNS. Its gene exists in three common isoforms: ApoE2, ApoE3, and ApoE4. Among these, ApoE4 is implicated as a genetic risk factor for late‐onset AD. Studies suggest that ApoE4 is less efficient in clearing Aβ, and the impaired clearance leads to Aβ accumulation, ultimately contributing to neurotoxicity and neuronal dysfunction.^37^ Additionally, ApoE4 has been implicated in heightened tau pathology, another key feature of AD. This finding suggests a potential role for ApoE4 in multiple neurodegenerative pathways, not just Aβ clearance. The presence of the ApoE4 allele is also associated with earlier disease onset and greater disease severity in individuals diagnosed with AD.^38^

Tau protein acts as a microtubule‐associated protein (MAP) that binds to microtubules, promoting their assembly and stabilization. It becomes abnormally hyperphosphorylated in AD and disrupts its ability to bind to microtubules, destabilizing them. The detachment of tau proteins from microtubules disrupts intracellular transport and results in neuronal dysfunction and impaired brain activity, leading to macroscopic atrophy of the brain.^21^ The hyperphosphorylated tau proteins then clump together, forming NFTs within neurons. These NFTs disrupt essential transport within neurons, hinder communication between them, and potentially damage them directly.^39^ Kinesin‐1, a molecular motor protein that is involved in microtubular cargo transports, is essentially responsible for intracellular transport. In AD, the accumulation of tau aggregates on microtubules blocks the movement of kinesin‐1, resulting in impaired transport. The role of kinesin‐1 dysfunction in AD still needs to be explored. In light of recent evidence, it is observed that reducing kinesin‐1 heavy chain (KIF5B) expression or complete knock out in cells, as well as tau transgenic mice have shown a significant decrease in the level of tau accumulation and stability. It is hypothesized that the tau interacts directly with KIF5B, through its microtubule‐binding region, which inhibits the ATPase activity of motor proteins.^40^

Glycogen synthase kinase‐3β (GSK‐3β) is involved in various cellular processes and its activity is tightly regulated to prevent excessive phosphorylation of tau in neurons. In AD, various factors like Aβ plaques and genetic predisposition can lead to dysregulation of GSK‐3β activity.^41^ This dysregulation causes GSK‐3β to become hyperactive, leading to excessive phosphorylation of tau protein at multiple sites and contributing towards disease progression. Further, studies suggest ApoE4 might directly interact with tau protein, altering its conformation and making it more susceptible to hyperphosphorylation.^42^, ^43^

**FIGURE 3:**
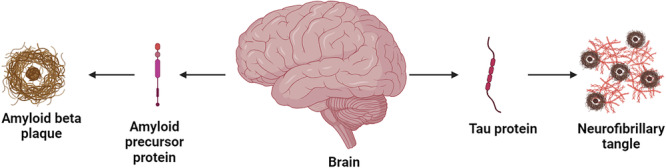
The degenerative cascade in Alzheimer's disease. It begins with the buildup of abnormal proteins like Aβ plaques and tau tangles, which cause damage to brain cells and disrupt communication between them, leading to memory loss and other cognitive impairments. [Color figure can be viewed at wileyonlinelibrary.com]

Aβ plaques appear initially in the hippocampus and entorhinal cortex areas which are vital for memory and learning. This initial damage contributes to early memory decline. NFT formation is minimal but may begin in these same regions. Aβ plaques further spread to the temporal and frontal lobes of the neocortex, while NFT pathology worsens, affecting the hippocampus, amygdala, and temporal lobe. This wider damage leads to more significant cognitive decline and memory impairment. In the latter stage, Aβ plaques become widespread throughout the neocortex, impacting frontal, parietal, and occipital lobes. NFT pathology becomes severe, encompassing most neocortical regions. This extensive neurodegeneration results in profound cognitive decline, language difficulties, and impaired motor function ^44^, ^45^ (Figure 3).

The amyloid cascade hypothesis indicated that the unusual aggregation of Aβ peptide and Aβ plaque development instigated the formation of NFTs and made neurons more vulnerable to neurotoxicity and death.^46^ Before the formation of Aβ plaques and NFTs, the emergence of soluble Aβ aggregates precipitates synaptic, dendritic, and neuronal degeneration, alongside significant dysfunction in neurotransmitter systems such as ACh and glutamate within the CNS.^47^ The exact cause of cholinergic neurodegeneration remains a mystery, but multiple factors are associated. Aβ plaques, a hallmark of AD, appear to directly harm cholinergic neurons or indirectly trigger inflammation that damages them. NFTs may disrupt vital processes within these neurons, leading to dysfunction and death. Chronic inflammation in AD further contributes to cholinergic neurodegeneration. Additionally, oxidative stress and glutamate dysregulation can overstimulate and damage these neurons, leading to cell death.^47^, ^48^, ^49^ According to prior research, a deficit in the cholinergic system may precede the development of the early histopathological hallmarks of AD, even before the onset of clinical symptoms.^50^ Glutamate, a critical neurotransmitter for learning and memory, is increasingly recognized as a potential contributor to AD. In excessive concentrations, glutamate can lead to excitotoxicity, a phenomenon characterized by the overstimulation of neuronal glutamate receptors. This excitotoxicity triggers a cascade of cellular events that culminate in neuronal damage and even cell death.^51^, ^52^ Mitochondria are responsible for ATP production through oxidative phosphorylation. In AD, impaired functioning of mitochondria leads to increased reactive oxygen species production that contributes to oxidative stress. This oxidative stress further disrupts mitochondrial function by damaging mitochondrial DNA and proteins, creating a vicious cycle that exacerbates neuronal dysfunction and cell death. Studies have shown elevated levels of oxidative stress markers and impaired mitochondrial function in AD brains, highlighting the potential significance of this pathogenic pathway in AD progression.^53^ Autophagy is essential for clearing toxic protein aggregates like Aβ and tau in AD. Autophagy impairments, often caused by lysosomal issues and genetic factors, lead to harmful protein buildup, which worsens neurodegeneration. Transcription factor EB (TFEB) and transcription factor binding to IGHM enhancer 3 (TFE3) are nuclear proteins that control the activity of genes involved in autophagy. Enhancing autophagy holds therapeutic promise for AD, as it may reduce neuroinflammation and improve protein clearance, though precise regulation.^54^ Peroxisome proliferator‐activated receptor alpha (PPARα) is a transcription factor that initiates autophagy and enhances TFEB function, thereby reinforcing the autophagy‐lysosomal pathway (ALP).^55^

Sirtuin 1 (SIRT1) is a deacetylase that promotes cell survival, reduces neuroinflammation, and regulates autophagy and Aβ degradation, which helps to protect neurons against AD‐related damage. Forkhead box O3a (FOXO3a) is a transcription factor regulated by SIRT1 that influences cellular stress responses, autophagy, and oxidative stress management.^56^ Thus targeting the pathophysiology related to autophagy is beneficial for the treatment of AD. In AD, the extracellular accumulation of Aβ aggregates, including toxic Aβ_1–42_ fibrils and mutant forms like Iowa and Dutch variants, disrupt neuronal function. Additionally, impairment of the ALP hampers the clearance of these aggregates, exacerbating their toxic effects. These Aβ aggregates interfere with cellular processes, leading to neuronal damage and death. The dysfunction of ALP contributes to the inability of neurons to degrade and recycle toxic proteins, amplifying the disease's progression.^57^

## 4 MOLECULAR TARGETS FOR AD TREATMENT

The search for effective treatments for AD necessitates the exploration of diverse molecular targets. While some of these targets possess known inhibitors, others remain under investigation to identify suitable ligands for therapeutic intervention.^58^

### 4.1 Beta‐site APP cleaving enzyme 1 (BACE‐1)

BACE‐1 is an aspartate protease that consists of a catalytic dyad composed of Asp32 and Asp228 residues present at the active site.^59^ In an unbound state, BACE‐1 adopts an energetically favorable “open‐flap conformation,” which is stabilized hydrogen bonds. The substrate binding induces conformational flexibility that adopts a “flap‐closed conformation.”^60^ These residues facilitate the proteolytic cleavage of APP at the β‐secretase cleavage site, leading to the release of soluble APPβ and the generation of Aβ peptides.^61^ Blocking Aβ production at the active site of enzymes like BACE‐1 reduces pathogenic Aβ_42_ levels and potentially slows neurodegeneration. Thus, combining active site inhibition of the enzyme with other therapies may yield synergistic benefits. However, the inhibition of BACE‐1 activity has faced challenges, including off‐target effects and toxicity issues, which have hindered their clinical translation.^62^

### 4.2 γ‐secretase

γ‐secretase is also an aspartate protease that consists of four subunits viz. “nicastrin,” “presenilin,” “presenilin enhancer 2” and “anterior pharynx‐defective 1.” The complex has 170 kDa molecular weight with an additional 60 kDa molecular weight derived from nicastrin glycosylation, reaching up to a total size 230 kDa with 19 trans‐membranous segments. It belongs to the family of intra‐membrane cleaving proteases that consists of aspartyl protease, zinc metalloprotease, site‐2 protease family, and serine protease. γ‐secretase is a multi‐subunit enzyme complex having proteolytic activity and plays a vital role in the generation of Aβ.^63^

### 4.3 Butyrylcholinesterase (BuChE)

BuChE is a hydrolase that is responsible for hydrolyzing esters of choline.^64^ Degeneration of the basal forebrain cholinergic system is an indication of AD.^65^, ^66^, ^67^ Studies have found that the biochemical properties of BuChE were changed in neurodegenerative diseases like AD. Due to the loss of neurons, there is a shortfall of ACh and AChE levels expressed in a high amount which was responsible for the reduction of neurotransmitters and its enzyme.^68^ In the cortical region, BuChE level is increased during AD, which is responsible for neuritic plaques and NFTs formation.^68^, ^69^

### 4.4 Calcium‐permeable AMPA receptors (CP‐AMPARs)

AMPA receptors (AMPARs) are one of the fastest ionotropic glutamate receptors regulated by excitatory neurotransmitters in the CNS.^70^, ^71^ It is involved in the regulation of CP‐AMARs in electrophysically produced synaptic plasticity and could be a therapeutic target for AD patients and other neurodegenerative diseases. During long‐term potentiation induction, CP‐AMPARs are employed from perisynaptic pools to contribute to boosting synaptic Ca^2+^.^72^, ^73^ Some researchers suggest that CP‐AMPAR is involved in the onset of synaptic pathology and formation of AMPAR and thus it is a therapeutic target for AD and other neurodegenerative diseases.^74^, ^75^

### 4.5 Calcitonin gene‐regulated peptide (CGRP)

CGRP plays an important role as a potent vasodilator. It is known as a neurotransmitter in the CNS, which contains 37 amino acids.^76^, ^77^ It is distributed in different parts of the brain like the hypothalamus, ventromedial nucleus of the thalamus, amygdala, gray matter, hippocampus, and dentate gyrate.^78^ CGRP also helps in improving learning and memory processing.^79^

### 4.6 Phosphodiesterase (PDE)

PDE consist of a group of enzymes that control the rate of cyclic adenosine monophosphate (cAMP) and cyclic guanosine monophosphate (cGMP) hydrolysis and also contain 11 types of protein family members.^80^, ^81^ In brain regions like the hippocampus and cortex striatum, PDE isoforms play a crucial role in the hydrolysis of cGMP^82^, ^83^ and intracellular signaling cascades. Studies suggest PDE2A, PDE5 as well as PDE9 are involved in memory formation.^84^, ^85^

### 4.7 Muscarinic and nicotinic ACh receptors (mAchR)

mAchR are Ach receptors located at various sites of the CNS. They are G protein receptor complexes present on the cell membranes of several neurons.^86^ They exhibit some vital functions like central cholinergic transmission mediating motor control, learning and memory process.^87^ These receptors are divided into five subtypes M_1_–M_5_, among which M_1_‐type mAChRs are located in the hippocampus and cerebral cortex and play an important role in functional impairment, memory, and learning in AD.^88^

These cholinergic deficiencies have become an important feature of AD and can be reversed by cholinergic activation. Several research suggest that M_1_ type of mAchR stimulates dephosphorylation of tau in PC12 cells, which is responsible for the alteration of hyperphosphorylation of tau protein and NFT pathology.^89^ mAchR subtypes facilitate a variety of presynaptic and post‐synaptic actions in hippocampus regions. The activity of M_1_ AChRs has been reported to reduce AD symptoms and restore brain functions. Some processes promote αAPP and reduce hyperphosphorylated tau, as well as counteract the hypocholinergic effects caused by Aβ. An M_1_ allosteric candidate, designed by GlaxoSmithKline, 77‐LH‐281, showed higher therapeutic potential and better CNS penetration. Two M_1_ selective agonists, VU0357017 and VU0364572, were selected from the Vanderbilt Center for Neuroscience Drug Discovery and tested on cell lines and animal models, and shown to be effective in a variety of parameters. Some preliminary trials with muscarinic agonists have been carried out that improve cognitive function in patients, but trial results cannot justify the hypothesis of AD.^26^ However, nicotinic neuronal receptors also physiologically respond to Ach and express the α7 and α4β2 subtypes. In AD patients, compound AF267B was found to have excellent pharmacokinetics and can cross the blood‐brain barrier (BBB) after oral administration, while AF102B, AF150(S), and AF267B have neurotropic effects, and promote non‐amyloid protein APP and Aβ in AD. It reduces the formation of amyloid plaque by decreasing the signaling ability of the receptors, which leads to a decrease in cholinergic activity.^90^ Based upon both muscarinic and nicotinic receptors, some of the first M_1_ agonists also failed after entering clinical trials because they were nonselective. EVP6124, an α7 nicotinic receptor agonist developed by Elan Pharmaceuticals, is currently in phase II trials and shows good results in AD patients when given alone and in combination with AChE inhibitors.^58^

### 4.8 Tau protein

Tau is a MAP that plays a vital role in the assembly and stability of the microtubules and maintains cell integrity. They are found in normal phosphorylated soluble form primarily in axons.^91^ The tau protein is hyperphosphorylated in AD and forms insoluble intracellular NFTs in neurons. This condition disturbs the normal synaptic plasticity and causes neurodegenerative changes. GSK‐3β, Tau kinase 1 and cyclin‐dependent kinase 5 (CDK5) are some of the major enzymes involved in hyperphosphorylation of tau.^92^ Thus, inhibition of the GSK‐3β brings down the hyperphosphorylation of tau protein and hence, it has been considered to be another beneficial therapeutic alternative. Many reputed pharmaceutical organizations like Eli Lilly, Roche, and Glaxo Smith Kline have tried and tested many small molecules as GSK‐3β inhibitors. Maleimide derivatives, oxadiazole, pyrimidine thiazolidine‐diones derivatives, benzimidazole, imidazopyridine, and quinolones are some of the most common molecules which have been recognized for the purpose and have shown positive results *in silico* and further in‐vitro assays.^93^ Source and functions of AD therapeutic targets were summarized in Table 1.

**TABLE 1:** Source and functions of AD therapeutic targets.

No.	Name of target	Source	Functions	References
1	β‐Secretase	Astrocyte	Formation of Aβ	[59]
2	Butyrylcholinesterase	Basal forebrain	Neuritic plaques and neurofibrillary tangles	[94]
3	γ‐Secretase: Presenilin I	Medial temporal lobe cortex	Formation of Aβ	[95]
4	CP‐AMPARs	Hippocampus	Boost synaptic Ca^2+^	[96]
5	Calcitonin gene‐regulated peptide regulated peptide	Hypothalamus, ventromedial nucleus of the thalamus, amygdala, gray matter, hippocampus and dentate gyrates	Neurotransmitter	[97]
6	Phosphodiesterase (PDE)	Hippocampus, Cortex stratum	Hydrolysis of CGMP	[98]
7	Muscarinic acetylcholine receptor (mAchR)	Hippocampus	Hyperphosphorylation of tau protein	[99]
8	Dopamine 2 receptor	Central nervous system	Aβ plaques	[100]
9	Gama aminobutyric acid A receptor	Cerebral cortex, temporal lobes, frontal and occipital lobes	Neurotransmitters in cerebral cortex	[101]
10	Nuclear factor E2 related factor‐2 (Nrf2)	Temporal lobe, microglia and astrocytes	Antioxidant, maintaining redox balance and eliminating damage proteins	[102]
11	Gamma‐secretase metabotropic glutamate receptor	Cortical and hippocampal	Releasing of Ca^2+^	[103]
12	Parkinson disease protein pus 7 (DJ‐1/PARK7)	Hippocampus	Antioxidant, molecular chaperon, protein degeneration and transcriptional regulation	[104]
13	N‐mycdownstream‐regulated gene 2	Astrocytes, glia cells	Differentiation, cell proliferation, and cell apoptosis	[105]
14	Serotonin 5‐HT6 receptor	Cortical	Improving cognition dysfunction, synaptic plasticity	[106, 107]
15	Protein tyrosine phosphatase 1B (PTP1B)	Hippocampal, microglial	Learning, memory, endoplasmic reticulum, stress, regulation of synapse dynamics, and microglial‐mediated neuroinflammation	[108]
16	Monoamine oxidase B (MAO‐B)	Astrogila, hippocampus, cerebral cortex, and astrocyte	Metabolism of monoamine neurotransmitter	[97]
17	NAD(P)H Quinone oxido reductase 1		Oxidative stress, abnormality in redox balance	[109]
18	Neurotrophic receptor tyrosine kinase 1	Basal forebrain nucleus basalis (NB)	Development of CNS and PNS, promotion of cell survival	[110]
19	Amyloid protein precursor (APP)	Hippocampus, olfactory bulb	Formation of Aβ	[111]
20	Peroxisome proliferator activated receptor‐γ	Astrocytes and microglial cells	Lipid storage, adipocyte differentiation, and lipid storage and glucose homeostasis	[112]
21	C‐C chemokine receptor type‐5	Cortical neurons, hippocampal	Recruitment of leukocytes to Inflammatory sites	[113]
22	Angiotensin receptor	Astrocyte, neurons, microglia and cerebrovascular endothelial cells	Anti‐inflammatory compounds and synaptic signaling	[114]
23	Non‐amyloid‐beta/A4 protein	Neocortex, hippocampus, olfactory striatum, thalamus, and cerebellum	Amyloidogenesis and plaque formation	[115]
24	c‐Jun N‐terminal kinases	Cortex hippocampus and cerebellum	Neuronal apoptosis, neural tube defects, and oxidative stress	[116]
25	Triggering receptor expressed on cells myeloid cells 2	Myeloid	Amyloid pathology	[117]

Abbreviations: Aβ, amyloid β; CP‐AMPARs, calcium‐permeable AMPA receptors; CGMP, cyclic guanosine monophosphate; CNS, central nervous system; PNS, peripheral nervous system.

## 5 RECENT AD THERAPEUTICS

Recent advancements in AD therapeutics emphasize both symptom management and addressing underlying pathological mechanisms. Lecanemab (Leqembi) is a monoclonal antibody approved in 2023 that targets Aβ plaques. It has shown efficacy in reducing amyloid deposits in early‐stage AD but poses risks such as amyloid‐related imaging abnormalities (ARIA) and infusion‐related reactions.^118^ Emerging immunotherapies, including donanemab and gantenerumab, also aim to clear Aβ plaques and attenuate tau‐related pathology, with ongoing evaluations for their long‐term cognitive benefits.^119^ Some innovative small molecules, such as F‐SLCOOH, have shown promise in preclinical studies for their dual role in detecting and targeting Aβ aggregates while enhancing autophagy pathways, offering both diagnostic and therapeutic potential.^57^ As the Aβ‐oligomer‐targeted fluorescent probe, F‐SLOH shows significant therapeutic promise in treating AD. In vivo studies using 5XFAD and 3XTg‐AD mouse models revealed that F‐SLOH inhibits Aβ aggregation and reduces levels of Aβ plaques, AβO, and tau aggregates. It also enhances the ALP to clear APP and tau metabolites. Additionally, F‐SLOH reduces neuroinflammation, mitigates synaptic deficits, and improves memory and cognitive function in these models.^120^ Tau protein therapies, including inhibitors of tau aggregation and tau‐targeting vaccines, seek to address the NFTs characteristic of AD. Neuroprotective agents, including BACE inhibitors and neurotrophic factors, play a vital role in enhancing neuronal resilience and maintaining synaptic functionality, complementing other therapeutic strategies for AD. Celastrol, derived from *Tripterygium wilfordii*, enhances autophagy and lysosomal biogenesis by activating TFEB. In AD models, it reduces phosphorylated tau aggregates, improving cognitive function. By promoting the TFEB‐mediated ALP, celastrol shows promise as a novel therapeutic agent for AD and tauopathies.^121^

Overexpression of APP facilitates its interaction with Fe65 protein via the Fe65‐PTB2 domain, triggering Aβ secretion. Hence, the disruption of the APP‐Fe65 interaction emerges as a therapeutic strategy. Engineered exosomes carrying corynoxine‐B, an autophagy inducer, successfully targeted APP‐expressing cells, induced autophagy, and improved cognitive function in AD mice.^36^ In another recent study, caudatin, derived from *Cynanchum otophyllum*, was found to bind to PPARα, enhancing ALP expression, promoting Aβ and phospho‐Tau clearance, and improving cognitive function in AD mice.^55^

## 6 RECENT CLINICAL FAILURES

**FIGURE 4:**
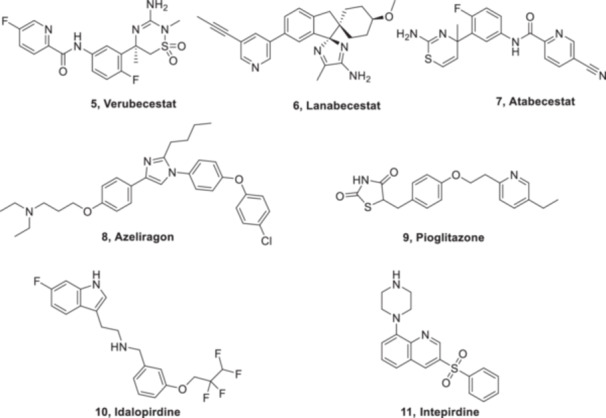
Recent clinical failure for Alzheimer's disease (AD). Despite early optimism, these treatments were unable to ameliorate or reverse the disease, highlighting the challenges in developing successful therapies for AD.

Despite the significant unmet medical need in AD, the number of drugs tested in clinical trials remains concerningly low. Very few drugs, that is, only 244, were investigated for AD in clinical trials between 2002 and 2012 according to clinicaltrials.gov.^122^ Out of the tested drugs, only memantine (**4**) was successful in clinical trials and ultimately FDA‐approved with a success rate of 0.4%. Despite exploring promising targets like those targeting the Aβ hypothesis viz. BACE1 inhibitors, anti‐Aβ antibodies, and other approaches (receptor for advanced glycation end products (RAGE), PPAR, 5‐hydroxytryptamine‐6 (5‐HT_6_)), most drugs tested in phase III clinical trials for AD failed. β‐secretase is one of the enzymes responsible for the amyloidogenic processing of APP to Aβ, and acts as a key player in the progression of AD. Three BACE inhibitors viz. Verubecestat (**5**, Merck), Lanabecestat (**6**, AstraZeneca & Eli Lilly), and Atabecestat (**7**, Janssen) have failed in clinical trials. Verubecestat has been shown to effectively reduce levels of toxic Aβ species in the blood, cerebrospinal fluid (CSF), and brain tissue. This effect has been observed not only in animal models but also in patients with AD.^123^ However, in early 2017, the EPOCH trial of Verubecestat involved patients with mild to moderate AD. The drug successfully lowered levels of Aβ in the CSF and reduced brain amyloid plaque buildup, but it did not slow down the worsening of AD symptoms in these patients.^124^ The APECS trial, specifically designed for patients with prodromal AD, was terminated early due to lack of efficacy. In this trial, the 40 mg dose of Verubecestat was not only ineffective but also worsened cognitive decline and increased adverse effects in patients. These findings, coupled with the negative results from the EPOCH trial in mild‐to‐moderate AD, cast doubt on the potential of Verubecestat as an effective treatment for AD.^125^ In mid‐2018, two additional clinical trials were initiated on Lanabecestat, namely AMARANTH, focusing on early AD, and DAYBREAK‐ALZ, targeting mild AD. However, both trials were abruptly halted. The treatment offered no benefit in slowing mental decline or daily activities. It did increase reports of psychiatric issues, weight loss, and hair color changes compared to a placebo.^126^ Atabecestat, an oral BACE‐1 inhibitor, initially showed promise by lowering Aβ levels in the CSF in japanese and Caucasian populations.^127^ However, both a phase II safety trial and a phase III efficacy trial were halted due to increased liver enzymes (hepatotoxicity) in patients taking Atabecestat.^128^ Thus, targeting BACE‐1 appears unlikely to yield fruitful outcomes. Solanezumab, an engineered antibody designed to target a specific form of Aβ in the brain, initially showed promise. Early studies phase II clinical trial indicated increased levels of unbound Aβ in the CSF, suggesting potential benefit.^129^, ^130^ However, larger phase III trials involving mild to moderate AD patients (EXPEDITION 1‐3) ultimately failed to demonstrate any significant improvement in cognitive function.^131^, ^132^ While some initial data suggested a possible benefit in mild AD (EXPEDITION 1), this wasn't confirmed in subsequent trials (EXPEDITION 2 & 3). These disappointing results cast doubt on Solanezumab's effectiveness as a treatment for AD. Azeliragon (**8**, vTv Therapeutics) targets a different pathway in AD. It is a small molecule designed to inhibit a receptor called RAGE, which is linked to inflammation and found in higher levels in AD patients. Aβ is a known ligand for RAGE and is thought to be responsible for promoting Aβ influx into the brain and disrupting BBB integrity.^133^ A phase II clinical trial investigated compound Azeliragon **(8)** and found that 18 months of treatment led to significant cognitive improvement in patients with mild AD.^134^ Disappointingly, a subsequent phase III study called STEADFAST was terminated in mid‐2018 due to a lack of efficacy. The results of this trial remain unpublished.^135^ Pioglitazone (**9**), an existing antidiabetic drug, has been repurposed for AD due to its function as a PPAR‐γ agonist.^136^ Prior research suggests that insulin resistance worsens tau phosphorylation and Aβ plaque deposition.^137^ Thus, PPAR‐γ agonists like Pioglitazone may offer a potential benefit in AD by reducing inflammation and amyloid plaque burden.^137^, ^138^ A small study observed improved blood flow to specific brain regions and enhanced cognitive function in patients with mild AD, suggesting potential benefits.^139^ Building on these findings, Zinfandel and Takeda Pharmaceuticals initiated a larger phase III clinical trial named TOMORROW in 2013. This trial had two key goals. The first objective was to determine if Pioglitazone could be an effective treatment for patients experiencing mild cognitive impairment (MCI) caused by late‐onset AD. The second objective aimed to assess a new genetic test's ability to identify individuals at risk of developing MCI, potentially allowing for earlier intervention strategies in AD management.^140^ Reports in early 2018 indicated that the TOMORROW trial did not yield positive results and was terminated. Furthermore, the results remain unpublished, leaving the future of Pioglitazone for AD treatment uncertain. Another promising in vivo study investigated Idalopirdine (**10**) developed by Lundbeck & Otsuka. This molecule acts by antagonizing the 5‐HT_6_ receptor, potentially influencing neurotransmitter activity and leading to positive effects on cognitive function.^141^, ^142^ However, it is important to note that earlier phase II trials of Idalopirdine yielded mixed results, not showing a significant cognitive improvement on its own.^143^ Interestingly, another study explored a combination of Idalopirdine with donepezil, which showed significant improvement in cognitive function compared to using donepezil alone.^144^ These findings suggest that Idalopirdine's potential benefits may be more pronounced when combined with other therapies. The phase III trials, named STARSHINE, STARBEAM, and STARBRIGHT, were carried out to investigate the efficacy of Idalopirdine as an add‐on therapy to existing AChE inhibitors, viz. donepezil, rivastigmine, or galantamine, in patients with mild‐moderate AD for 6 months. Unfortunately, none of these trials demonstrated cognitive improvement with the combination therapy.^145^ Intepirdine (**11**), a drug designed to block 5‐HT₆ potentially linked to memory and learning, showed initial promise in early clinical trials for AD. However, development stalled after phase II (small‐scale testing) as a larger phase III trial (with more patients) failed to demonstrate significant cognitive improvement^146^ (Figure 4).

## 7 CURRENT REPURPOSED DRUGS AND THEIR CLINICAL TRIAL FOR THE TREATMENT OF AD

Drug repurposing offers a faster and cost‐effective avenue in the journey of finding therapeutics targeting AD. However, significant challenges persist. The complex and heterogeneous nature of AD poses a substantial hindrance to researchers in identifying efficacious pharmaceutical agents. Furthermore, the repurposed drugs possess a risk of the potential hazard of unforeseen adverse reactions, requiring additional clinical evaluations despite established safety profiles. Moreover, the complexities of acquiring intellectual property rights further hinder the repurposing of existing medications. Regardless of these obstacles, the advancement of computational methodologies and enhanced understanding of disease pathology are driving forces for the exploration of drug repurposing as a promising strategy within the realm of AD.

There is a growing concern about the heterogeneity within AD. The distinction of AD cases into different subgroups based on etiology, pathophysiology, and clinical presentation is crucial. CSF biomarkers, particularly Aβ, tau, and ubiquitin levels, have been employed to stratify AD patients into five subgroups.^147^ Each subgroup exhibits a unique nonclinical and clinical profile, suggesting potentially divergent underlying disease mechanisms within the broader spectrum of AD. The intranasal administration of insulin through ApoE4 carriers showed distinct efficacies in contrast to non‐carriers for AD.^148^ In the past decade, significant advancements have been achieved in AD research, particularly in the areas of genetic risk factors, brain imaging, and CSF and plasma biomarkers.^149^ These developments should be applied for classifying patients in future clinical trials of AD which would enhance the precision and efficacy of therapeutic interventions. Another aspect of the current investigation for the therapy of AD is based upon the drug repurposing of the FDA‐approved molecule for other conditions. Some groups of drugs, such as antidiabetic drugs and anti‐inflammatory drugs, are under exploration for their probable efficacies for the treatment of AD.^150^ The repurposing approach is highly intriguing and has gained substantial support from many investigators due to its unconventional methodology for AD drug development. This approach has also received endorsement from the National Institutes of Health (NIH). A reason for using this approach is that the off‐target effects of numerous drugs also indirectly target the pathways associated with the mechanisms of AD. In 2021, an investigators meeting was organized by the National Institute on Aging entitled “Translational Bioinformatics Approaches to Drug Repurposing and Combination Therapy Development for AD/ADRD,” which presented various motivating progress related to this field. The previous clinical trials with individually employed drug candidates have failed to exhibit significant efficacies against AD. Interestingly, network‐based approaches such as retrospective studies are used to evaluate a large number of human data could be to identify the benefits of the combination of such drugs in preventing and treating AD as well as dementia.^151^ The list of FDA‐approved drugs that underwent clinical trials is presented in Table 2.

**TABLE 2:** FDA‐approved drugs that have been repurposed for the treatment of AD.

Drug	Mechanism of action	Molecular pathways	Dose	Outcome of trial	Clinical trial information
Pioglitazone	PPAR‐γ agonist	Insulin signaling pathway	15–45 mg daily	Did not show significant improvement in AD	Phase 3, NCT02284906
Atorvastatin	HMG‐CoA reductase inhibitor (statin)	Cholesterol metabolism pathway	10–80 mg daily	No significant cognitive benefit observed	Phase 2, NCT00024531
Riluzole	Glutamate release inhibitor	Glutamatergic pathway	50 mg twice daily	Showed potential neuroprotective effects	Phase 2, NCT01703117
Lithium	Mood stabilizer, GSK‐3β inhibitor	Glycogen synthase kinase pathway	150–600 mg daily	Showed potential to slow cognitive decline	Phase 2, NCT02129348
Valproate	Anticonvulsant, histone deacetylase inhibitor	Epigenetic regulation pathway	250–1500 mg daily	No significant benefit, increased adverse effects	Phase 3, NCT00071721
Simvastatin	HMG‐CoA reductase inhibitor (statin)	Cholesterol metabolism pathway	20–80 mg daily	No significant cognitive improvement observed	Phase 2/3, NCT00053599
Metformin	AMPK activator, insulin sensitizer	Insulin signaling pathway	500–2000 mg daily	Ongoing, potential metabolic benefits	Phase 2, NCT04098666
Exenatide	GLP‐1 receptor agonist	Insulin signaling pathway	2 mg weekly	Showed potential cognitive benefits	Phase 2, NCT01255163
Liraglutide	GLP‐1 receptor agonist	Insulin signaling pathway	0.6–1.8 mg daily	Improved brain glucose metabolism	Phase 2, NCT01843075
Nicotinamide	Vitamin B3, NAD+ precursor	NAD+ metabolism pathway	250–1000 mg daily	Showed potential to improve cognitive function	Phase 2, NCT03061474

Abbreviations: AMPK, AMP‐activated protein kinase; cGMP, cyclic guanosine monophosphate; GLP‐1, glucagon‐like peptide‐1; GSK‐3β, glycogen synthase kinase 3 β; HMG‐CoA, 3‐hydroxy‐3‐methylglutaryl‐coenzyme A; NAD, nicotinamide adenine dinucleotide; PDE5, phosphodiesterase type 5; PPAR‐γ, peroxisome proliferator‐activated receptor gamma.

## 8 MULTI‐TARGET APPROACH FOR DRUG DEVELOPMENT IN AD

Developing treatments for AD is complex because the disease has multiple contributing factors and its exact mechanisms are still being unraveled. This necessitates unconventional approaches and strategies beyond typical drug development.^152^ One promising strategy in AD treatment involves designing drugs with multiple “active functional groups.” These drugs, often called multi‐targeted drugs, can possess several functional groups, allowing them to target multiple molecular pathways involved in AD progression. Traditional drug development often focuses on drugs with a single, specific target or those with various nonspecific effects. Interestingly, these nonspecific activities might prove beneficial if they inadvertently target pathways relevant to AD pathogenesis. In the context of AD, with its multiple contributing factors, nonspecific drug candidates might even be preferable to highly specific ones. This is because their nonspecific actions could potentially target additional mechanisms underlying AD that have not yet been fully identified.^153^

During the last three decades, several researchers have furnished many possible molecular mechanisms underlying AD. Although a complete understanding of the mechanisms of the disease is still a long way from being reached. The data obtained from brain imaging, bioinformatics, and biomarkers studies have furnished fruitful facts that can assist in increasing the efficacies of the multi‐target strategy for the treatment of AD. Several multifactorial approaches along with their pathologies, molecular mechanisms, and clinical symptoms of AD patients could be adopted for the designing of novel compounds.

Precision medicine is an idea for the treatment of individual patients as per their specific epigenetic, environmental, genetic, metabolic, and social profiles. The execution of human genomic studies at feasible costs for individual genome screening, coupled with brain imaging and various biomarkers will make a precision medicine‐based approach achievable for treating AD. This kind of approach is exclusively useful in multi‐target therapy because the targets can be planned based on the patients individually.^154^, ^155^

GSK‐3β, a key protein kinase, has emerged as a potential target in AD research. This enzyme plays a crucial role in phosphorylating tau, a protein whose abnormal accumulation is linked to neurodegeneration in AD.^156^ Hence, inhibitors of GSK‐3β have been explored and exposed to clinical trials for the management of AD.^157^ The inefficacy observed in clinical trials targeting GSK‐3β for AD may be attributed to a compensatory mechanism known as redundancy. Multiple redundant protein kinases, exceeding twelve in number, and including several tau‐specific kinases, exhibit the capability of phosphorylating tau protein in both in vitro and in vivo environments. This redundancy underscores the potential insufficiency of solely inhibiting GSK‐3β to impede tau phosphorylation and achieve a demonstrable clinical benefit in AD patients.^158^ Thus, exclusive GSK‐3β inhibition is not enough to inhibit abnormal hyperphosphorylation of tau.^158^ Nonspecific inhibitors of GSK‐3β also inhibit additional tau kinases along with GSK‐3β and may notably inhibit atypical tau hyperphosphorylation. GV‐971 is a natural compound derived from marine algae that targets multiple biological processes. It has been approved by the China Food and Drug Administration for the treatment of AD. GV‐971 is believed to work by inhibiting the clumping of Aβ protein, reducing inflammatory responses triggered by astrocytes in the brain, and promoting a healthy gut microbiome.^159^, ^160^, ^161^ All these mechanisms are thought to be involved in the development of AD. Synthetic efforts have targeted bifunctional compounds, each moiety harboring activity against a discrete signaling pathway implicated in AD.^162^, ^163^ This objective can be achieved through the strategic linkage of pharmacophoric moieties derived from distinct bioactive entities.^164^ Thus, each pharmacophore of the new hybrid drug can maintain its capacity to interconnect with their particular sites on the targets and generate numerous specific pharmacological responses. This approach can abolish simultaneous multiple drug administration with potentially distinct degrees of pharmacokinetics, metabolism, and bioavailability and make a streamlined therapeutic regimen. In light of this idea, it is worth investigating a single bifunctional active AD vaccine immunotherapy against both Aβ and tau pathologies. The screening of natural products with multiple actions and mechanisms against various insults involved in AD could be another fruitful multi‐target strategy. Several natural products, such as chelerythrine,^165^ chalcone,^166^ coumarin,^167^ huprine,^168^ curcumin,^169^ rhein,^170^ berberine,^171^ and derivatives of resveratrol,^171^ have shown such potential and warrant further studies for AD drug development.^172^ The new drug, GV‐971, a natural product, belongs to this category.^173^

Combination therapies targeting multiple aspects of AD offer another promising approach to treat the disease by addressing its complex mechanisms. Single‐drug therapies often fail to effectively treat AD because they target only one pathway, while AD likely involves multiple pathways in each patient. Thus, according to the multifactorial AD hypothesis, more than one drug with concurrent treatments targeting a well‐defined mode of action might have significant efficacy. On the other hand, a molecule that can regulate or modulate several molecular pathways associated with neurodegeneration and progression of AD is also potential. Steen et al and Liu et al reported that insulin signaling is dysregulated in the AD brain due to impaired brain glucose metabolism, which is likely involved in neurodegeneration in AD.^174^, ^175^, ^176^ The restoration of insulin signaling in the brain through intranasal insulin administration or conventional oral drug delivery is currently under investigation as a potential therapeutic approach for enhancing insulin sensitivity in the treatment of AD.^177^ The restoration of brain proteins through the process of O‐GlcNAcylation represents another promising strategy within this approach. O‐GlcNAcylation involves the modification of proteins by the addition of β‐N‐acetylglucosamine (GlcNAc) to the hydroxyl groups of serine or threonine residues.^178^ O‐GlcNAcylation modulates several pathways implicated in AD pathogenesis, including the processing of APP, tau phosphorylation, synaptic integrity, and insulin signaling.^179^, ^180^, ^181^, ^182^, ^183^ In AD, a reduced O‐GlcNAcylation of both tau and global proteins is observed in the brain.^180^, ^181^ The in vivo mice models displayed that the restoration of the brain O‐GlcNAcylation pathway could improve cognitive function in AD.^184^, ^185^ Further, the neurotrophic agents can initiate neurogenesis through several pathways and have vast potential for the treatment of AD. Compound P021, a neurotrophic peptidergic compound, that prevents deficiencies associated with neuronal plasticity and neurogenesis, was found to counteract Aβ plaque formation, reverse cognitive impairment, oppose tau pathologies, and intensely reduce the mortality rate in transgenic mice 3xTg‐AD.^186^, ^187^

## 9 INTERVENTIONS FOR THE PREVENTION OF AD

The strategies for the prevention of AD are two types, primary and secondary prevention. The key idea of primary prevention is lowering risk variables for AD with a plan of action like lifestyle modification, supplementation, treatments of co‐morbidity, and multi‐factorial interventions.^188^ On the other hand, secondary prevention begins with mechanism‐based interventions to prevent cognitive symptoms with the expectation of treating basic pathophysiology.^189^

### 9.1 Primary prevention interventions

Primary prevention is intended for specific lifestyle interventions such as changes in exercise and diet, metabolic risk factors, cognitive inducement, and social commitment.^190^ Several studies revealed that yoga, meditation, and naturopathy provide a calm state of mind and have some influence on AD.^191^ Cardiovascular risk factors such as hypertension and hyperlipidemia are found to be associated with AD and dementia. In Europe, the project Syst‐Eur correlating systolic hypertension with vascular dementia indicated a significant reduction in systolic blood pressure, by at least 20 mmHg and a goal of under 150 mmHg, reducing the incidence of dementia.^192^ This intervention was found to lower 50% incidence of dementia, along with AD dementia incorporated as a subcategory. The Framingham heart study revealed that the incidences of dementia have reduced over the past 30 years which might be due to improvements in the treatment of cardiovascular disease over time.^193^ Various studies concluded that hyperlipidemia is one of the primary risk factors for the progression of AD, but the fact remains debatable.^194^ Two trials, viz. the Prospective Study of Pravastatin in the Elderly at Risk (PROSPER) and Heart Protection Study (HPS) (NCT00939822), have assessed the outcome of statins in AD.^195^ The trials signify no favorable effects of statins in the prevention of AD or cognitive decline.^196^ Diabetes appears to be a central point for the prevention of AD. A multi‐site randomized study was conducted called Action to Control Cardiovascular Risk in Diabetes Trial with Memory in Diabetes (ACCORD‐MIND). When the group with complete glycemic control of hemoglobin (HbA_1c_ less than 6%) was compared with the standard of care with hemoglobin (HbA_1c_ between 7% and 7.9%), there was no distinction found in the cognitive scores and brain volume (NCT00182910).^197^ However, early pilot studies of insulin treatment in MCI and AD subjects showed favorable effects on cognitive function (NCT00438568).^198^ Currently, a phase II/III clinical trial (SNIFF: Study of Nasal Insulin to Fight Forgetfulness) is underway in amnestic MCI and mild AD patients to find out the outcome of intranasal insulin on changes in CSF biomarkers, cognitive decline, and brain volume loss (NCT01767909). Metformin and pioglitazone are two insulin‐sensitizing agents that have been advanced into clinical trials of AD (NCT02432287; NCT00982202).^199^ Nonpharmacological interventions like vitamin supplements, diet, exercise, and cognitive stimulation have also been studied in AD prevention trials. Mediterranean diet, loaded with fruits and vegetables, incorporated with olive oil and fish is the most promising diet intervention. The Three‐City Study on the mini‐mental status examination showed that participants who stuck to the Mediterranean diet had a slower rate of cognitive decline.^200^ Other study, that is, Mediterranean‐Intervention for Neurodegenerative Delay (MIND), revealed that the Mediterranean diet minimizes the risk of AD up to 50% and provides protective effects which continue till later time points even when the recommended diet was not followed strictly.^201^

There have been several studies exploring the relation between physical activity and AD, where the inverse association has been implicated despite the moderate quality of evidence.^201^ Generally, it is suggested that physical activities in combination with diet modification or cognitive and social stimulation may be more helpful in reducing the risks of AD.^201^ A randomized controlled trial, Fitness for the Aging Brain Study (FABS), was conducted on participants with memory complain but no dementia. It displayed a moderate improvement in cognition at 18 months in subjects who were doing a planned physical activity compared to the control group (ACTRN12605000136606).^202^ When a Mediterranean diet is combined with physical activity there is a significant fall in AD incidence. For participants with a peak score for both Mediterranean diet and physical activity, the ratio hazard for AD was only 0.65.^203^ Besides physical activity, the effects of training on cognition have also been assessed. The robust study still now is the Advanced Cognitive Training for Independent and Vital Elderly (ACTIVE) trial that furnished powerful evidence for the advantageous effects of cognitive intervention in cognitive impairment prevention (NCT00298558).^204^ A more comprehensive observation is collected regarding the usage of supplements such as *Gingko biloba*, vitamin E, and omega‐3 fatty acids for the prevention of AD. There is no confirmation yet suggesting any useful effects of vitamin E on preventing MCI transformation into AD or improving cognitive function of MCI or AD patients.^205^ Nonetheless, vitamin E and memantine combination therapy in subjects of AD may retard functional decline in MCI or AD patients by 19% per year over the 4‐year time period (NCT00235716).^206^, ^207^ The clinical studies of *Ginkgo biloba* extract, known as EGb 761, Ginkgo Evaluation of Memory (GEM) study, and GuidAge (NCT00010803) (NCT00276510) showed no beneficial effects in AD prevention.^208^, ^209^ Omega‐3 fatty acids have a complex association with AD, where 7 out of the 11 observational studies showed positive outcomes, but none of the four clinical trials showed any benefit for the treatment or prevention of dementia.^210^ A trail was conducted on omega‐3 fatty acids in combination with docosahexaenoic acid (MIDAS) to evaluate improvement of memory (NCT00278135).^211^ Multifactorial interventions targeting vascular and lifestyle risk factors have been tested in AD prevention trials such as the Prevention of Dementia by Intensive Vascular Care study (PreDIVA), the Finnish Geriatric Intervention Study to Prevent Impairment of Cognition and Disability (FINGER; NCT01041989), the Multidomain Alzheimer Preventive Trial (MAPT), and the Multimodal Preventive trial for AD (MIND‐ADMINI; NCT03249688).^212^ These ongoing multi‐centric trials highlight the significance of international collaboration and study design standardization.

### 9.2 Secondary prevention interventions

Secondary prevention trials have been started through worldwide collective efforts since 2011. Five large trials are included in secondary prevention, such as Autosomal‐Dominant AD (APIADAD), API APOE4 Trial, the Dominantly Inherited Alzheimer Network‐Trials Unit (DIANTU), the Anti‐Amyloid Treatment in Asymptomatic Alzheimer's Disease (A4) trial, and TOMMORROW Trial. The API DIANTU and A4 have already formed a group called the Collaboration for Alzheimer's Prevention to keep up systematic communication about the design of the study and validate its outcome. Among five trials, four trials are regularly conducted by the active group, testing the amyloid‐based hypothesis to ensure and provide equivalent and relevant cognitive results.^190^ Under secondary prevention trials, several questions are to be conveyed including guidelines based upon safe supervision of subjects for ARIA,^189^ intervention timing for maximum welfare of prevention and inventive cognitive results for approval of drugs under investigation for early AD with monitoring of their post‐approval.^213^

## 10 CONCLUSION

AD, the most common form of dementia, affects the geriatric age group. Depending on the stages and severity of the disease, it affects the brain and results in neurodegeneration. AD is a complex interplay of several pathological processes, and as such, its actual cause remains unknown. The early focus has largely been the amyloid hypothesis that states the formation of β‐amyloid peptides in plaques as the trigger for the disease, although further evidence indicates a more complex course of events. Tauopathies are related to the abnormally high phosphorylation of tau proteins that accumulate as tangles that promote neuropathology. The data shows that targeting amyloid plaques and tau tangles pathology have not provided a cure for the disease. Further, neuroinflammation, oxidative stress, and synaptic dysfunction also play their part in worsening the condition. The involvement of these systems highlights the need for complex therapy to address these multiple dysfunctions at once. These assertions imply that therapeutic interference in AD should be organized before the manifestation of frank clinical signs. There are several biomarkers, such as amyloid positron emission tomography scans and markers of CSF, that can predict the risk of people developing AD at an early stage.

Current treatments focus on symptom management rather than halting disease progression. Recently, several therapeutic strategies have emerged, including FDA‐approved drugs like lecanemab that target Aβ plaques and emerging monoclonal antibodies such as donanemab and gantenerumab, which aim to reduce tau‐related pathology. However, these therapies often come with side effects like ARIA. Additionally, small molecules like F‐SLOH are being explored for their dual diagnostic and therapeutic potential. F‐SLOH targets Aβ aggregates, promotes autophagy, and reduces neuroinflammation, providing hope for early‐stage AD treatment. Another promising approach involves enhancing the ALP to clear toxic proteins. Caudatin, a compound from *Cynanchum otophyllum*, activates PPARα and TFEB, leading to the degradation of Aβ and tau aggregates. Celastrol, a compound from *Tripterygium wilfordii*, has been shown to activate TFEB‐mediated autophagy, promoting the clearance of phosphorylated tau and improving cognitive function in AD mouse models. Despite these promising advancements, many therapies remain in experimental stages and require further validation through large‐scale clinical trials.

Risk‐modifying treatments started at an early stage of the disease process could halt the neurodegenerative process. Studies and clinical trials should therefore involve designing better drugs with the clear purpose of targeting neurodegeneration in the preclinical phase. Due to the heterogeneity of AD, one has to move toward precision medicine. Furthermore, higher genetic risks like ApoE ε4 allele increase the liability of developing AD in the patient. Besides, associated factors such as environmental and lifestyle factors, that is, vascular disease, diabetes, and head injury, have significant impacts on disease risk. This way, clinicians can assign individualized treatment due to the capability to contribute patients' genetic data, medical records, and such factors as diet and exercise. Effective treatments are urgently required to alleviate the symptoms and arrest the progression of the disease. Using customized multi‐targeted and biomarker‐guided strategies, we can achieve both effective and safe preventive therapies, based on the disease characteristics of each patient.

## AUTHOR CONTRIBUTIONS

Tapas Kumar Mohapatra was responsible for conceptual design, data collection, data analysis, and original draft preparation; Reena Rani Nayak participated in original draft preparation; Ankit Ganeshpurkar contributed to review and editing; Prashant Tiwari and Dileep Kumar conducted supervision, project administration, and review and editing.

## CONFLICT OF INTEREST STATEMENT

The authors declare no conflicts of interest.

## ETHICS STATEMENT

Not applicable.

## Data Availability

No new data set was generated in this review. All data cited and analyzed in this review are obtained from publicly available research articles, which are appropriately referenced in the manuscript. Readers may access the original data through the respective publications.
